# The Off-Target Effects, Electrolyte and Mineral Disorders of SGLT2i

**DOI:** 10.3390/molecules25122757

**Published:** 2020-06-15

**Authors:** Giuseppe Cianciolo, Antonio De Pascalis, Lorenzo Gasperoni, Francesco Tondolo, Fulvia Zappulo, Irene Capelli, Maria Cappuccilli, Gaetano La Manna

**Affiliations:** 1 Department of Experimental Diagnostic and Specialty Medicine (DIMES), Nephrology, Dialysis and Renal Transplant Unit, S. Orsola Hospital, University of Bologna, 40100 Bologna, Italy; giuseppe.cianciolo@aosp.bo.it (G.C.); lorenzo.gasperoni3@gmail.com (L.G.); francesco.tondolo@studio.unibo.it (F.T.); fulvia.zappulo@studio.unibo.it (F.Z.); irene.capelli@unibo.it (I.C.); maria.cappuccilli@unibo.it (M.C.); 2 Nephrology and Dialysis Unit, V Fazzi Hospital, 73100 Lecce, Italy; depascalis.a@libero.it

**Keywords:** SGLUTi, diabetic kidney disease, CKD, CKD-MBD

## Abstract

The sodium-glucose cotransporter 2 inhibitors (SGLT2i) are a relatively new class of antidiabetic drugs that, in addition to emerging as an effective hypoglycemic treatment, have been shown to improve, in several trials, both renal and cardiovascular outcomes. In consideration of the renal site of action and the associated osmotic diuresis, a negative sodium balance has been postulated during SGLT2i administration. Although it is presumable that sodium and water depletion may contribute to some positive actions of SGLT2i, evidence is far from being conclusive and the real physiologic effects of SGLT2i on sodium remain largely unknown. Indeed, no study has yet investigated how SGLT2i change sodium balance in the long term and especially the pathways through which the natriuretic effect is expressed. Furthermore, recently, several experimental studies have identified different pathways, not directly linked to tubular sodium handling, which could contribute to the renal and cardiovascular benefits associated with SGLT2i. These compounds may also modulate urinary chloride, potassium, magnesium, phosphate, and calcium excretion. Some changes in electrolyte homeostasis are transient, whereas others may persist, suggesting that the administration of SGLT2i may affect mineral and electrolyte balances in exposed subjects. This paper will review the evidence of SGLT2i action on sodium transporters, their off-target effects and their potential role on kidney protection as well as their influence on electrolytes and mineral homeostasis.

## 1. Introduction

Sodium-glucose co-transporter 2 (SGLT2) inhibitors are a class of anti-hyperglycemic drugs approved for the treatment of type 2 diabetes. Members of this class approved by FDA and EMA for clinical use, respectively in the USA and Europe, are canagliflozin, dapagliflozin, empagliflozin and ertugliflozin. The current understanding of mechanisms by which SGLT2 inhibitors exerts renal and cardiovascular protection entail that these drugs block renal reabsorption of glucose by inhibiting SGLT2 transporter, the primary sodium-coupled glucose transporter in the proximal tubules, promoting glycosuria and lowering blood glucose. Inhibition of glucose and sodium reabsorption leads to increased delivery of sodium itself to the macula densa, activating the tubuloglomerular feedback and promoting afferent arteriolar constriction, which in turn decreases intra-glomerular pressure and hyperfiltration which is detrimental in progressive nephropathy [1].

The decline in the glomerular filtration rate (GFR) could also be secondary to an increment in proximal tubule hydrostatic pressure following a decrease in reabsorption of sodium and therefore water uptake [2].

Indeed, the lowering of glomerular hyperfiltration would translate into the reduction of albuminuria, a marker of diabetic nephropathy, contributing to reduce the progression of kidney disease.

Furthermore, the loss of glucose contributes to weight loss which, together with the depletion of sodium and water, favors the reduction of blood pressure and blood volume.

These are considered the most likely factors that lead to cardiovascular protection on the basis of natriuretic and osmotic effects following SGLT2 inhibition [3,4,5,6,7].

Although these SGLT2i properties are potentially relevant both for cardiovascular and renal protection in diabetic patients, evidence about their effective mechanisms of action are far from being fully understood. Indeed, it is known that genetic knockout of SGLT2 in mice lowers blood glucose levels in streptozotocin (STZ)-induced diabetes mellitus and prevents glomerular hyperfiltration but the absence of SGLT2 doesn’t avoid the STZ-induced increase in kidney weight or in markers of renal growth, injury, inflammation, and fibrosis [8].

Recently, numerous experimental evidence has identified several alternative pathways, not necessarily linked to the handling of sodium, on which the action of the SGLT2i is carried out and that could be the basis of renal and cardiovascular protection [8,9,10,11].

Finally, although clinical data suggest that SGLT2i are safe, little attention has been dedicated to their effects on other electrolytes than sodium. Indeed, while the natriuretic effect is always reported, these drugs may also modulate potassium, magnesium, phosphate, and calcium urinary excretion.

The aim of this review is a detailed analysis of the SGLT2 inhibitor mechanisms on sodium handling, associated electrolyte abnormalities and off-target effects.

## 2. Classic Pathways Involved in Nephroprotection Mediated by SGLT2i

### 2.1. Effect of SGLT2i on Sodium Homeostasis

In healthy human kidneys, the proximal tubule (PT) reabsorbs between 60 and 80% of sodium load which glomeruli filter every day. The SGLT2 transporter plays a significant role in this process, reabsorbing one Na^+^ ion for every glucose molecule from the lumen of the early PT. Sodium and glucose, not reabsorbed in the early proximal tubule via SGLT2, reach other sodium transporters such as SGLT1 or Na^+^/H^+^ exchanger-3 (NHE3)-dependent, localized at more distal segments of the PT [9]. However, in healthy subjects this pathway is underused because over 90% of the filtered glucose is reabsorbed via SGLT2 transporters.

In diabetes, the higher expression and activity of SGLT2 and the full recruitment of SGLT1 cause an increased sodium reabsorption in the proximal tubule which means a reduced sodium uptake at the macula densa [1,10]. This pathway activates tubulo-glomerular feedback by a reduced synthesis of vasoconstrictive molecules acting on afferent arteriola, leading to increased intraglomerular capillary hydrostatic pressure and finally hyperfiltration [1]. SGLT2 inhibition has the opposite effect: sodium not absorbed proximally via SGLT2 pass distally and SGLT1 activity in the S2/3 segments of the proximal tubule can only partly compensate for this [11]. (NHE3)-dependent sodium uptake is also unable to fully compensate loss following SGLT2 inhibition. Together, these changes result in an increased delivery of sodium to the macula densa and a fall in intraglomerular pressure. However, available data in type 2 diabetes patients suggest that enhanced natriuresis is transient. Furthermore, in our opinion, tracing the benefits of the drug exclusively to a natriuretic effect due to SGLT2 inhibition may be a reductive interpretation. Indeed, we have to consider that along the tubule, at the most distal segments, a series of compensatory processes take place which ultimately tend to reabsorb available intraluminal sodium thus finally reducing natriuresis.

An evaluation aimed at defining the effects of SGLT2i on sodium balance cannot be separated from an analysis of its interaction with transporters located along the entire renal tubule. Several sodium transporters may be activated to increase sodium uptake in response to the mild natriuresis caused by SGLT2 inhibitors. In humans, 10 different sodium–hydrogen exchangers [12] have been identified [11]. These antiporters exchange sodium for protons in order to restore pH against intracellular accumulation of acid. In the kidney sodium–hydrogen exchangers are found in the proximal tubule (NHE3), in the macula densa [12] and thick ascendent limb of Henle (NHE4). NHE3, as mentioned above, exists in the proximal tubule and is responsible for both 30% of the sodium reabsorption and 70% of filtered sodium bicarbonate. Glucose seems to exert a bimodal effect on proximal tubule NHE3; while normoglycemia stimulates NHE3 transport activity, high glucose concentrations may inhibit this exchanger. Since SGLT2 and NHE3 co-localize in the rat renal proximal tubule, it has been hypothesized that they may be physiologically associated. SGLT2 inhibition decreases activity and function of NHE3, therefore contributing to natriuresis.

### 2.2. Effect of SGLT2i on Nephroprotection

SGLT2i lowers glomerular hyperfiltration and may reduce the progression of kidney disease in a manner that is independent of both glycemia and glycosuria.

This property of SGLT2i seems, albeit indirectly, confirmed by the post-hoc analysis of a trial subgroup with CKD stages 3B to 4, demonstrating an insignificant reduction in glucose levels and eGFR whilst albuminuria and blood pressure were reduced [13].

Another relevant player in the renal sodium handling is the isoform sodium–hydrogen exchanger 2 [12], placed on the macula densa cells. NHE2 participates in Na transport as well as it is also involved in macula densa salt-sensing and renin control. This a crucial point if we consider that cells of macula densa are ultimately the target of SGLT2i.

Although there is no direct evidence, Kimura speculated that SGLT2 inhibitors may act as loop diuretics, determining the conditions for an impairment on the activity of Na-K-2Cl cotransporters (the transport mechanism in the loop of Henle) [14]. Because proximal tubules are highly permeable to water, the tubular fluid in proximal tubules remains isotonic to blood. As a result of the isotonic reabsorption of Na and chloride (Cl), the ratio of Cl to water, that is, 4:8 at the proximal tubular origin (glomerular filtrate), gradually changes to 3:7 and then to 2:6 [14]. However, since glucose acts as a non-resorbable substance, the total solute concentration remains constant and isotonic, but the intraluminal concentrations of Na and Cl decrease gradually, and this low Cl concentration fluid is delivered to the loop of Henle. The reabsorption in the loop of Henle, located after the proximal tubules, is usually enhanced in a compensatory manner when proximal tubular reabsorption is inhibited. However, this compensatory increase in tubular reabsorption does not take place when SGLT2 is inhibited. The transport mechanism in the loop of Henle is composed of Na-K-2Cl co-transporters, suggesting that Cl plays an essential role compared with other electrolytes such as Na or K. A decrease in Cl intraluminal tubular concentration, following SGLT2 inhibition, results in a significant reduction of tubular reabsorption in the loop of Henle, probably because the number of necessary Cl molecules is twice the number of Na or K. The diuretic action of SGLT2i could be due to the inhibition of reabsorption in the loop of Henle, rather than that in proximal tubule; in this way, they would indeed act as loop diuretics.

This an important point if we consider that the cells of macula densa are ultimately the target of SGLT2i. The macula densa is a group of cells, similar to those present in the loop of Henle, located at the end of the cortical thick ascending limb, forming a juxtaglomerular apparatus-glomerular complex. These cells play a pivotal role in sensing changes in tubular fluid composition, generating and sending signals to the juxtaglomerular apparatus that control renal blood flow and GFR through tubuloglomerular feedback and renin release. Although the mechanism is not fully defined, salt sensing by the macula densa involves apical NaCl transport pathways, including the furosemide-sensitive Na-K-2Cl cotransporters (NKCC2) and NHE2 isoform. In cells of macula densa, the NKCC2 activity could be impaired, as mentioned, by the decrease in Cl intratubular concentration, following SGLT2 inhibition, and this pathway may also contribute to the inhibition of glomerulotubular feedback.

Similar implications about a possible impairment of glomerulotubular feedback involves the simultaneous use of the loop diuretics and SGLT2i.

In a post-hoc analysis from the EMPA-REG OUTCOME, the authors underline how—since loop diuretics inhibit Na-K-2Cl co-transporters—it is presumable that they block the entry of sodium and chloride into macula densa cells and consequently the glomerulotubular feedback [15]. Therefore, co-administration of loop diuretics could abolish the initial eGFR dip that is expected after the initiation of empagliflozin if tubuloglomerular feedback is inhibited as hypothesized. In this analysis, however, the eGFR dip with empagliflozin was still preserved in the presence of loop diuretics. Regardless, in the presence of Na-K-2Cl channel inhibition with loop diuretics, sodium and chloride ions may still activate tubuloglomerular feedback by entering into macula densa cells, possibly via the off-target effect of SGLT2i on other sodium transporter pathways such as the SGLT1 transporter and sodium–hydrogen exchanger.

A possible effect on glomerulotubular feedback mediated by SGLT1, localized not only in the proximal tubule but also in cortical thick ascending limbs of Henle and macula densa, is conditioned by the specificity for SGLT2 of different SGLT2 inhibitors.

SGLT2i inhibition of the family of NHE’s is speculative and much remains to be proven. In preclinical models, empagliflozin has been demonstrated to have 80% of the NHE1 inhibitory effect of cariporide in both rat and rabbit ventricular myocytes [16], however NHE1 is not located in the kidney. NHE-3 and NHE-4 are found in the proximal tubule and in the thick ascending limb of Henle, respectively.

The role of the isoform NHE2, found in the macula densa, where it participates in Na transport as well as the regulation of cell volume and intracellular pH, seems to be potentially relevant. Hanner et al. found that NHE2 is also involved in macula densa salt-sensing and renin control, and speculate that macula densa cell shrinkage is the likely cellular signal that activates renin release signaling [12].

However, it must be highlighted that the traditional framework about the effect of SGLT2iarises from several experimental (murine) models showing that SGLT2 inhibition elicits renal hemodynamic changes that attenuate glomerular hyperfiltration, thus demonstrating the important role of tubulo-glomerular feedback in early renal hemodynamic abnormalities diabetes related and lowers urine albumin excretion [17,18,19].

These findings have been confirmed in a study conducted by Cherney et al. in patients with type 1 diabetes [20] treated with empagliflozin: authors also demonstrated that the reduction of glomerular hyperfiltration was mediated by tubular-glomerular feedback mechanisms that induced afferent arteriolar vasoconstriction and increased renal vascular resistance [20]. Although the results of clinical trials are to be considered encouraging in terms of nephroprotection, there is a growing awareness that this effect cannot be attributed to a glucose-lowering action of these drugs. Indeed, regardless of the aforementioned considerations about the effective pathway and transporter involved in tubulo-glomerular feedback following sodium-glucose cotransporter-2 inhibitors, it remains unclear whether these renal hemodynamic changes can explain all the benefits of SGLT2i and the nephroprotection whatever the type of diabetes: type 1 or 2. Van Bommel et al. evaluated whether the renal hemodynamic effects of SGLT2 inhibitors observed in young adults with T1D and murine models of T1D held true in older adults with T2D. They found that dapagliflozin reduced intraglomerular pressure without increasing renal vascular resistance by lowering efferent arteriolar resistance, findings that potentially implicate different effects of SGLT2 inhibition on renal hemodynamic function in in older adults with T2D versus young adults with T1D [21].

Although there is no univocal explanation of these different features some considerations can be made. First, the different characteristics of the two cohorts beyond diabetes type: age, glycemic control, blood pressure, and renal function; second, the decline with age of the number of nephrons; third, the heterogeneity of histological picture underlying the diabetic kidney disease in older persons with T2D than in T1D.

## 3. Alternative Pathways Involved in Nephroprotection by SGLT2i

### 3.1. SGLT2i Sodium and Inflammatory Cytokines

**Figure 1:**
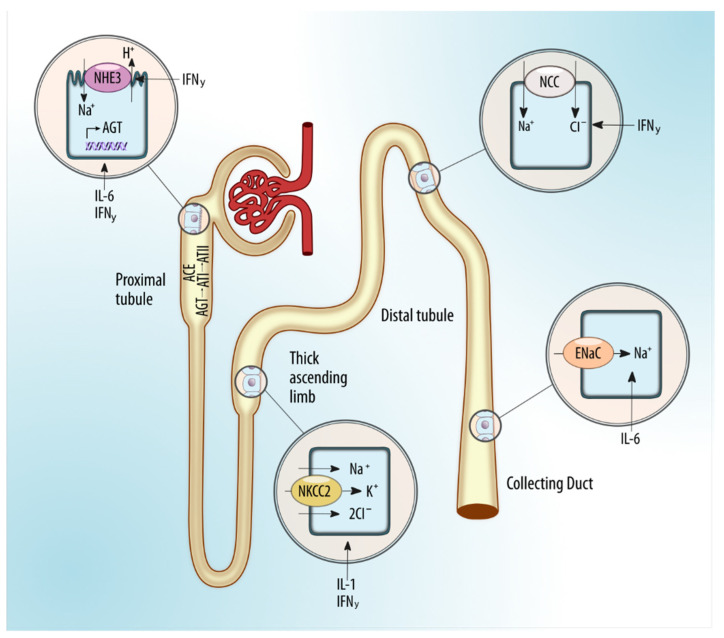
The complex interplay at renal tubule between sodium, tubular transporters, and inflammatory cytokines [22,23,24]. Abbreviations: AGT, angiotensinogen; ATI, angiotensin I; ATII, angiotensin II; ACE, angiotensin-converting enzyme; NHE3, sodium/hydrogen exchanger 3; IFN-γ, interferon-γ; IL-6, interleukin-6; NKCC2, sodium-potassium-two chloride cotransporter; NCC, sodium-chloride cotransporter; ENaC, epithelial sodium channel; IL-1, interleukin-1.

Inflammatory cytokines such as interleukin 6 [22], interleukin 1 [22], interferon-γ [23] and tumor necrosis factor α (TNFα) can affect sodium handling through changes in the expression of renal sodium transporters, renin angiotensin aldosterone system(RAAS) and nitric oxide [24] bioavailability [25]. IFN-γ positively regulates sodium/hydrogen exchanger 3 (NHE3) in the proximal tubule and NKCC2 and the sodium chloride co-transporter (NCC) in the distal tubule. Like IL-6, IFN-γ has been shown to increase angiotensinogen production from cultured renal proximal tubule cells, thus suggesting that IFN-γ may regulate sodium reabsorption through activation of the intrarenal RAS (Figure 1). In opposition, anti-inflammatory cytokines such as interleukin-10 induce a decrease in sodium transporter activity [23]. IL-1 and TNF-α have been shown to reduce endothelial nitric oxide synthase (eNOS) expression in the thick ascending limb, blunting NO production and enhancing sodium reabsorption, since NO upregulates NKCC2 expression. Moreover, interestingly, increased sodium has been shown to promote inflammatory cytokines and inhibit anti-inflammatory function of innate and adaptative immune cells.

This interplay between sodium handling and cytokines could have a role on hypertension, renal dysfunction, and diabetes. There is now compelling data denoting that diabetes includes an inflammatory component that is thought to be related to its main complications. Plasma concentrations of proinflammatory cytokines are high in diabetic patients and studies have shown that the concentrations of these substances increase with the progress of nephropathy [26]. Moreover, interestingly, increased sodium has been shown to promote inflammatory cytokines and inhibit anti-inflammatory function of innate and adaptative immune cells. In mice models, SGLT2i proved to be effective in lowering the release of inflammatory cytokines [24]. Human studies are still limited, however, in a cohort of fifteen diabetic patients, empagliflozin increased anti-inflammatory cytokines IL-10 [27].

### 3.2. Pleiotropic Effect of SGLT2i and Nephroprotection

The potential anti-inflammatory effect of SGLUTi is not only related to sodium handling which could ultimately influence, in a reciprocal manner, the balance between inflammatory and anti-inflammatory cytokines, but also to its involvement in several pathways linked to oxidative stress, inflammation, and autophagy. In diabetes, these processes are deranged and activate several pathways that lead to cell dysfunction and death as well as inflammation and fibrosis.

Autophagy is a self-degradative process that is important for promoting energy efficiency through ATP generation, and for the removal of misfolded or aggregated proteins and damaged cellular organelles. Thus, autophagy is generally thought of as a survival mechanism, although its deregulation has been linked to non-apoptotic cell death [28].

Diabetic kidney disease reveals an important impairment of adenosine monophosphate- activated protein kinase (AMPK) and sirtuin-1 (SIRT1) signaling, which may contribute to the development and progression of nephropathy. These enzymes lower oxidative stress and inflammation and stimulate autophagy; the efficiency of this degradative pathway is crucial for homeostasis of podocytes, tubular cells, mesangial and glomerular endothelial cells. The activation of AMPK preserve podocyte and renal tubular structure and glomerular function, whilst the overexpression of SIRT1 improves glomerular and renal tubulointerstitial injury in experimental models of diabetic nephropathy [29,30].

**Figure 2:**
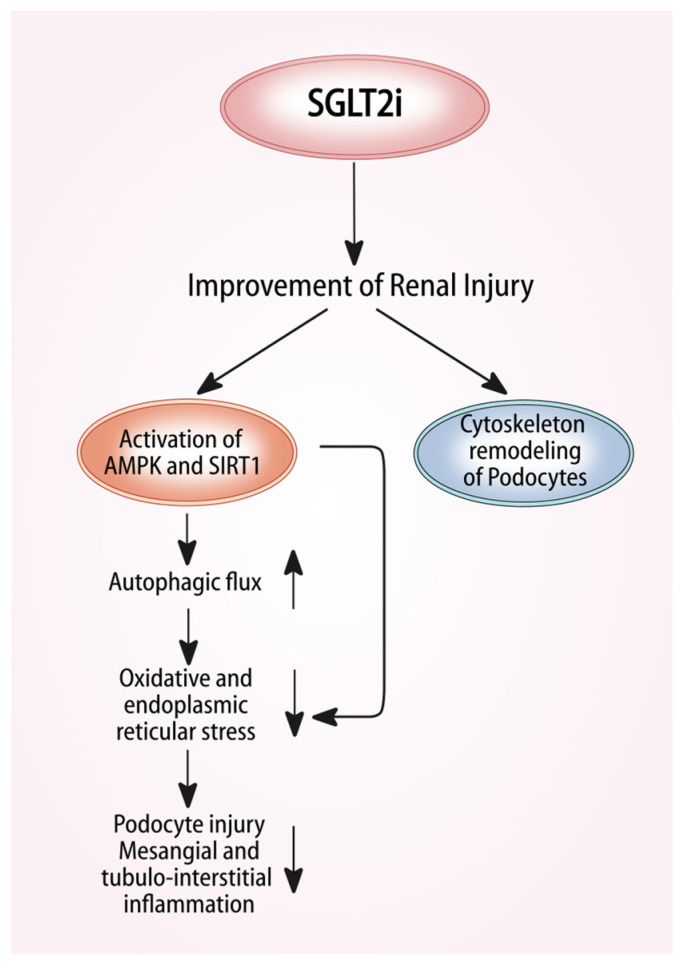
Pleiotropic effects of sodium-glucose cotransporter 2 inhibitors (SGLT2i) and nephroprotection: SGLT2i induce both AMPK and SIRT1, both stimulate authophagy, thus reducing cellular stress, glomerular and tubular injury [25,26]. *Abbreviations*: AMPK, adenosine monophosphate-activated protein kinase; SIRT1, sirtuin-1.

SGLT2 inhibitors induce both AMPK and SIRT1, and they have been shown to stimulate autophagy, thus reducing cellular stress, glomerular and tubular injury (Figure 2). AMPK and SIRT signaling is also closely linked to sodium transport mechanisms, regulating the intracellular sodium. The activation of AMPK and SIRT1 lead to down-regulation of NHE and the epithelial sodium channel (ENaC), whose activity is enhanced in diabetes [31], and in this way induce a low intracellular sodium content, improving the antioxidative defense systems [32]. SGLT2 inhibitors lead to enhanced AMPK/SIRT1 signaling thus increasing authophagy and tubuloglomerular feedback both underpinning the SGLUTi protective effects on diabetic kidney disease [33].

The protective effects of SGLT2 inhibitors are not only mediated by the activation of AMPK and SIRT1 signaling but could also be due to their direct action on podocytes. Cassis et al. used the mouse model of protein-overload proteinuria in which the loss of the permselective properties of the glomerular barrier to proteins is a major determinant for disease progression. Mice were studied for 23 days after starting bovine serum albumin [34] injection, a time in which they exhibit proteinuria and glomerular lesions but mild tubular damage. Treatment with dapagliflozin had a remarkable antiproteinuric effect, associated with amelioration of the glomerular lesions, similar to that observed with an ACE inhibitor. SGLT2 was highly expressed in the kidneys of mice after BSA injections and importantly, SGLT2 inhibition prevented the increase in blood glucose levels and corrected defective natriuresis in mice with protein-overload proteinuria. However, the natriuretic effect of dapagliflozin was not associated with changes in GFR, as measured at the end of the study on day 23 after starting BSA injections, suggesting that in this experimental setting, the improvement of proteinuria and glomerular lesions was unlikely the consequence of natriuresis-dependent glomerular hemodynamic function. The hypothesis is that in cultured podocytes dapagliflozin limits cytoskeletal remodeling induced by albumin load [34].

In recent years, evidence has accumulated suggesting that chronic hypoxia may be the primary pathophysiological pathway driving diabetic kidney disease and chronic kidney disease of other etiologies, and it was coined the “chronic hypoxia hypothesis” [35,36]. It has been speculated that an imbalance between oxygen delivery and oxygen demand, also known as “chronic hypoxia hypothesis” is an additional mechanism underlying CKD progression, whatever its etiology. Chronic hypoxia is sustained by the damage of capillaries that leads to a reduction of glomerular and post-glomerular blood flow [35].

This imbalance is further worsened in diabetes, where several mechanisms act, increasing ATP, and therefore increasing oxygen demand and simultaneously decreasing ATP generation.

In diabetes, increased renal ATP demand follows a) an enhanced renal sodium transport associated with glucose overload on proximal tubular cells; b) an increase in GFR due to the tubuloglomerular feedback mechanism. Decreased ATP generation is thought to be secondary to insulin resistance, therefore in diabetes, the ATP deficit and increased renal oxygen demand are associated with a reduction in oxygen delivery, due to hyperglycemia-related damage of the microvasculature [37,38].

Renal tissue oxygenation is the result of an accurate balance between oxygen delivery and oxygen demand. While oxygen delivery depends on renal perfusion, oxygen demand is mainly related to the ATP production required for the tubular reabsorption of filtered sodium. Several tubular transporters, including SGLT2, involve a further considerable oxygen demand since they use the electrochemical gradient settled by the active extrusion of sodium by the Na/K-ATPase (NKA) pump at the basolateral membrane to transport sodium. The production of ATP to drive the NKA pump is the primary source of oxygen requirement, as over 90% of ATP is generated through oxidative phosphorylation [37,39].

Hesp and coworkers have identified renal hypoxia a promising target for renoprotective effects of SGLUTi [40].

Several studies provide growing evidence for a beneficial effect of SGLT2 inhibition on renal oxygenation and tissue damage. In addition to the decrease in renal oxygen demand, SGLT2 inhibition is also able to increase oxygen supply by the attenuation of capillary rarefaction of peritubular capillaries [40].

### 3.3. SGLT2i Sodium and Cardiomyocytes

**Figure 3:**
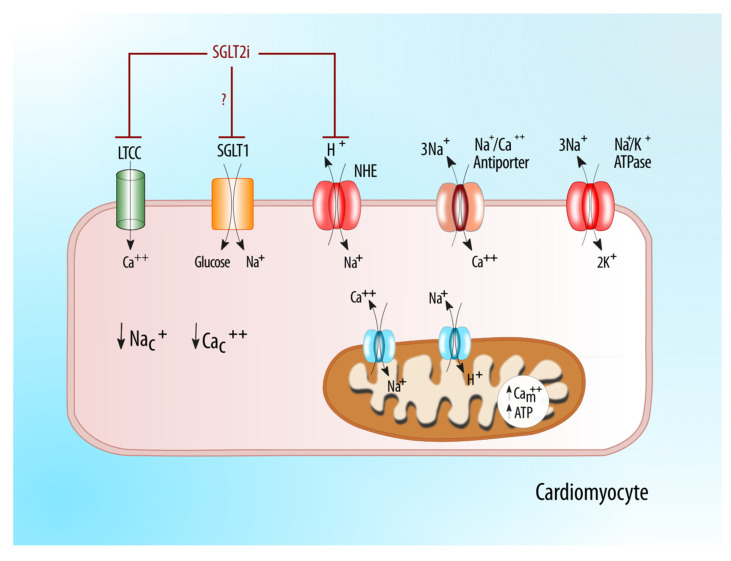
Direct effects of SGLT2ion the cardiomyocyte: inhibition of NHE1 on cardiomyocite could reduce Na and Ca intracellular concentration overload, the pathogenetic pathway of contractile failure [11,15]. *Abbreviations*: LTCC, L-Type Calcium Channels; NHE, sodium/hydrogen exchanger; Na_c_^+^, intracitoplasmatic sodium; Ca_c_^++^, intracitoplasmatic calcium; Ca_m_^++^, intramitochondrial calcium.

Sodium concentrations in the skin and muscles are reportedly positively correlated with the risk of cardiovascular diseases and blood pressure in chronic kidney disease [27]. Impaired sodium–hydrogen exchange has been demonstrated to have deleterious effects on cardiomyocytes by the increased intracellular concentration of sodium [22,41]. This increased intracellular sodium tendency has been well demonstrated in myocardial hypertrophy and heart failure states, both acute and chronic. The NHE family has homology in structure to the SGLT family in terms of a funnel shape for sodium transport [42]. A strong off-target effect of SGLT2i on the sodium–proton antiporter (exchanger) on the cell surface, and intracellular organelles could explain the wide-ranging effects of these agents. In preclinical models, SGLT2i have been demonstrated to have 80% of the NHE1 inhibitory effect of cariporide in both rat and rabbit ventricular myocyte [16]. The SGLT2 inhibition of NHE1 on cardiomyocytes could reduce intracellular concentrations of sodium, subsequently reducing calcium overloading which is a common pathophysiologic mechanism of contractile failure (Figure 3). Interestingly, chronic treatment with dapagliflozin significantly reduces sodium concentrations in the skin of type 2 diabetic patients. These data support the hypothesis that SGLT2 inhibitors decrease the sodium concentration in interstitial fluid. The reduction in intracellular sodium could be due to the inhibition of the above mentioned sarcolemmal Na^+^-H^+^ exchanger (the NHE hypothesis). NHE1 is the predominant isoform in the heart and vasculature, while NHE3 is expressed at the apical surface of renal epithelial cells where it co-localizes and functionally interacts with SGLT2. In patients with T2D and heart failure, the activity of NHE1/3 is markedly enhanced. This increase facilitates the accumulation of intracellular Na^+^ ([Na^+^]i), which stimulates the reverse activity of the Na^+^/Ca^2+^ exchanger (NCX) leading to an increase in [Ca^2+^]i and cardiomyocyte injury, facilitating mitochondrial Ca^2+^ extrusion to the cytoplasm and decreasing mitochondrial Ca^2+^ ([Ca^2+^]m). The reduction in [Ca^2+^]m impairs the Ca^2+^-induced stimulation of the Krebs cycle dehydrogenases and reduces ATP production and mitochondrial antioxidative capacity [11].

At this time, the physiologic impact and direct cardiac tissue effect of SGLT2i have not been fully assessed, but preclinical data suggest that they have protective effects on cell survival, function, and organ adaptation.

## 4. SGLT2 Inhibitors-Induced Electrolyte Abnormalities

### 4.1. Effect on Magnesium and Potassium Homeostasis

**Figure 4:**
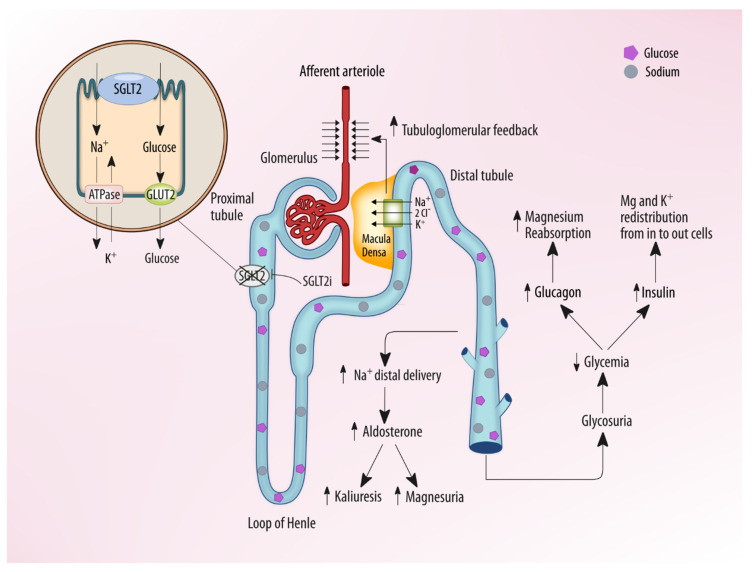
Effects of SGLT2i on serum electrolytes: SGLT2 inhibition promotes glycosuria, natriuresis, and osmotic diuresis, determining enhanced aldosterone activity with increased kaliuresis and magnesuria. Such effects are counterbalanced by an improvement in glycemic control with an elevation of serum glucagon and reduction of insulin, which favors redistribution of potassium and magnesium in cells from the intracellular space. The final effect is a potential low increase of serum potassium and magnesium levels.

Mild changes in serum potassium concentration during SGLT2 inhibitor therapy have been reported in several studies. Small increases in serum potassium have been reported, associated with high doses of canagliflozin but not with empaglifozin and dapaglifozin [43,44]. It is not clear whether this effect on potassium is caused by small transient changes in GFR or volume status. Indeed, a slight reduction in volume status leads to a reduction in sodium delivery to the cortical collecting duct in the distal nephron, potentially resulting in less sodium exchanged for potassium. Moreover, the decreased insulin concentrations observed with SGLT2 inhibitors administration, due to reduced glucose levels and to improvement in insulin resistance, could result in a redistribution of potassium out of the cells, thus increasing serum levels (Figure 4). However, even though the increase in serum potassium concentration is small, its careful monitoring is indicated in patients with renal impairment, or in patients on therapy predisposing to hyperkalemia, such as RAAS inhibitors [45].

Furthermore, some concerns have been raised regarding an increased risk of diabetic ketoacidosis (DKA) [46] in SGLT2 inhibitor-treated patients. In these cases, similarly with the classic DKA, an increased anion gap metabolic acidosis and increased risk for hyperkalemia is observed [46]. SGLT2i-induced DKA is, in most cases, accompanied with normal serum glucose levels (euglycemicDKA) and not with severe hyperglycemia [47]. The main metabolic alteration in SGLT2i-associated DKA seems to be the imbalance between insulin and glucagon serum levels. A glucosuria-mediated reduction in serum insulin levels in T2DM patients treated with SGLT2i might lead to increased lipolysis which, along with a reduction in serum glucose levels, induces an increased fatty acid oxidation in myocytes and hepatocytes leading to an increased production of acetyl-CoA, which then can be converted into ketones [48]. It should be mentioned that in most individuals, SGLT2i-associated DKA is asymptomatic, but in some, especially in the presence of predisposing factors such as a low reserve of insulin-secreting cells, a sudden decrease in insulin dose, hypovolemia, acute illness, surgery, or alcohol abuse, it may lead to symptomatic DKA.

SGLT2 inhibitors can also increase serum magnesium levels in diabetic patients [49]. Some different mechanisms are reported in the SGLT2 inhibitor-induced serum magnesium concentration increase but the precise mechanism is still unknown [50]. Among these, the attenuated magnesium wasting-induced hypomagnesemia associated with diabetes mellitus is one of the most likely—this increased magnesiuria in diabetes patients may be the effect of the reduced transient receptor potential ion channel 6 (TRPM6) activity in the distal convoluted tubules related to insulin resistance [51].

In fact, a down-regulation of TRPM6 channels resulting in hypermagnesiuric hypomagnesemia is reported in obese type 2 diabetic rats [52]. The improvement of insulin resistance observed after SGLT2 inhibitors administration is probably associated with a restored activity of TRPM6 and a consequently reduced magnesium excretion. Moreover, the increased glucagon concentrations commonly observed during SGLT2 inhibitor administration can affect magnesium homeostasis, since glucagon is associated with increased magnesium reabsorption in the distal renal tubules [53]. Furthermore, insulin induces a shift of magnesium from the plasma to the intracellular space, thus, its reduced levels may be followed by a redistribution of magnesium out of the cells to the extracellular space. Finally, SGLT2 inhibitors can increase aldosterone concentrations due to natriuresis and osmotic diuresis-induced hypovolemia, and it is well known that aldosterone may have a direct effect on magnesium transport, leading to increased magnesium excretion. In fact, spironolactone, an aldosterone inhibitor, can decrease renal magnesium wasting. In actuality, available clinical data have shown that RAS inhibition is transient and the plasma aldosterone level did not significantly change through treatment with an SGLT2 inhibitor [53]. This may be because aldosterone production is stimulated not only by Angiotensin II, but also by other factors, such as adrenocorticotropic hormone and potassium and the natriuretic action of SGLT2 inhibitors is usually transient. Thus, it seems that SGLT2 inhibitor administration ultimately leads to a small increase in serum magnesium levels. In the general population and in people with type 2 diabetes, a gradient of risk for cardiovascular disease has been observed across the normal range of serum magnesium, with concentrations at the higher end of the normal range associated with a lower risk of cardiovascular events [54,55]. In fact, the increase in serum magnesium may decrease the risk of cardiac arrhythmias, thus in part explaining some of the cardiovascular benefits associated with SGLT2 inhibitors.

### 4.2. Effect of SGLT2i on Calcium and Phosphate Homeostasis

Although data are still not conclusive, it is important to emphasize that a growing body of evidence suggests that the use of SGLT2i may have some effects on bone health and the mineral metabolism index [24]. Increased bone fragility and risk of fracture are features of both type 1 diabetes and T2D, and may be caused by poor bone quality and abnormal bone microarchitecture [56]. These features are likely the consequence of many common variables, including chronic hyperglycemia, tissue-specific accumulation of advanced glycation end-products (AGEs), abnormal insulin levels, chronic low-grade inflammation, sclerostin, wingless/integrated (Wnt) signaling pathways and FGF23-klotho axis [57,58,59].

Therefore, in diabetic patients, chronic low-grade inflammation as well as the factors involved in its onset can be expected to affect, besides cardiovascular health, bone cell activity and bone health [60,61]. Because of these assumptions, whatever the effects of SGLUTi on mineral metabolism, their action is carried out on bone tissue frequently altered by diabetes.

The results of the trials carried out so far, though reassuring, are probably still not enough to address the safety issue in high-risk subgroups of patients, such as those with chronic kidney disease (CKD). Indeed, the limited study follow-up (which ranged from six months to two years) and the heterogeneity of the case-mix and study design of different randomized controlled trials RCTs preclude a definitive answer on the impact of these compounds on a long-term outcome, such as the risk of fracture. In addition, some concerns arise from animal model studies that also support the notion that SGLT2i can potentially affect bone metabolism [57,58]. Mice treated with canagliflozin exhibited increased calciuria (because of the osmotic diuresis triggered by glycosuria) together with a rise in the serum levels of FGF-23 and PTH. Changes in gene expression (about an 11-fold increase) of renal CYP27B1 and sodium-dependent phosphate transporter 2A (about a 30% decrease) were also assessed [57,58]. Whereas the former is a key factor in vitamin D metabolism, the latter regulates renal phosphate handling.

Two studies have tried to define the pathways involved in changes of bone biomarkers associated with SGLT2i therapy. In a study carried out on healthy volunteers, treatment with canagliflozin increased serum phosphate by 16%, FGF-23 by 20%, and PTH by 25%, as well as decreased 1.25-dihydroxyvitamin D by 10%, compared with controls. The raise in serum phosphate after Canagliflozin administration follows a significant increase in renal tubular reabsorption of phosphate, whilst the increase in plasma FGF-23 was consistent with the increase in serum phosphorus, thus explaining the decrease in plasma 1,25-dihydroxyvitamin D [62]. The results of a secondary analysis of a crossover double blinded, placebo-controlled trial confirm the findings. In particular, De Jong and colleagues ascertained a significant increase in serum phosphate (9%), FGF-23 (19%), and parathormone PTH (16%) in a cohort of 31 patients with T2D with CKD stages 2 to 4 and albuminuria who were treated with dapagliflozin for six weeks. Remarkably, these changes were associated with a significant 12% decrease in 1,25-dihydroxyvitamin but no change on tubular reabsorption of phosphate and serum calcium [63]. It is plausible that SGLT2i entail an increased phosphate-dependent sodium reabsorption in the proximal tubule caused by decreased glucose-dependent sodium reabsorption. By inhibiting the cotransport of both Na^+^ and glucose, SGLT2i increase Na^+^ availability in the regions of the proximal tubule where NaPi-2a and NaPi-2c are placed and modulate renal phosphate handling. The elevated intraluminal Na^+^ concentration, in turn, increases the electrochemical drive to reabsorb phosphate. Higher serum phosphate induces the osteocyte synthesis of FGF-23 that inhibits renal expression of CYP27B1 and 1,25-dihydroxyvitamin D synthesis. Low calcitriol levels are, in turn, responsible for high PTH levels directly via reduced feedback inhibition, and indirectly by reducing dietary calcium absorption. This combined pattern of reduced 1,25(OH)2D and increased PTH and FGF-23 levels likely contribute to the increased fracture risk associated with SGLT2i.

However further efforts are needed to define some crucial mechanisms underlying these changes. In particular, we must ask: what are the variations of calcium excretion? While an increased urinary calcium has been reported in rats and mice treated with SGLT2i, in opposition, Blau and colleagues found that 24-h urinary calcium excretion was decreased two days after the 1,25-dihydroxyvitamin D levels nadir; this finding is joined with maximal increases in PTH. In healthy volunteers, an increase in serum phosphorus and FGF-23 and a lowering in 1,25(OH)D2 is only transient, and these biomarkers trend back to baseline values [62]. In contrast, the phosphate and PTH increase persists, and is larger in patients with impaired renal function, suggesting that SGLT2i may change mineral homeostasis [63]. An analysis of these topics may be relevant in the perspective of long-term exposure to these drugs, which appears all the more important considering that ongoing clinical trials are currently testing the efficacy and safety of SGLT2 inhibition in CKD patients with or without DKD [64].

## 5. Conclusions

The modulation of the renal sodium-glucose co-transporter 2 (SGLT2) provides us with the opportunity to effectively control glucose metabolism in diabetes. Clinical data from RCT suggest that these new compounds (Empagliflozin, Dapagliflozin, Canagliflozin) are safe and protect against renal and cardiovascular events. At any rate, these undeniable protective effects can only partly be traced back to a better metabolic control, improved natriuresis and water loss. Natriuresis is limited and no study has investigated how SGLT2i change sodium balance in the long term, and especially the real physiologic effects of sodium renal handling. Experimental studies suggest that many of the effects outside the glycemic control must be considered as off-target effects, which seem crucial in the interplay of SGLT2i with sodium. Moreover, little attention has been dedicated to the effects of these compounds on the renal handling of different electrolytes. While the natriuretic effect and osmotic diuresis are expected, these compounds may also modulate urinary chloride, potassium, magnesium, phosphate, and calcium excretion.

We can expect, in the future, that the use of SGLT2 inhibition for cardiorenal protection may extend beyond patients with diabetic kidney disease and may likely better explain the beneficial effects of these drugs on renal and cardiovascular outcomes.
